# Factors that influence beef meat production in Tanzania. A Cobb-Douglas production function estimation approach

**DOI:** 10.1371/journal.pone.0272812

**Published:** 2022-08-12

**Authors:** Cornel Anyisile Kibona, Zhang Yuejie, Lu Tian

**Affiliations:** 1 College of Economics and Management, Jilin Agricultural University, Changchun, Jilin, China; 2 Department of Agricultural Economics and Finance, Mwalimu Julius. K. Nyerere University of Agriculture and Technology, Musoma, Tanzania; University of Nairobi, KENYA

## Abstract

Beef meat production is the key to reducing poverty, achieving food security and nutrition, promoting exports, economic growth, and industrialization. Despite a large number of beef cattle, Tanzania continues to import beef meat and its contribution to GDP is low. Thus, this study used time-series panel data to analyze the beef meat industry in Tanzania from 1990 to 2019, with a particular focus on identifying the reasons and direction of the correlation between beef meat output and its determinants in the production processes. The study applied both descriptive statistics and the Cobb-Douglas production function model, using the Ordinary Least Squares (OLS) based estimator to analyze the data. Descriptive analyses revealed that Tanzania’s beef meat production increased by 283,871 tons (59.3%-a positive trend) between 1990 and 2019. This increase was accompanied by approximately 29.75%, 53.05%, and 42.42% increases in beef cattle yield (carcass weight (hg) per head, beef cattle inventory, and the number of beef cattle slaughtered, respectively). However, the analysis further revealed that a 2.8% decrease in beef cattle extraction (low harvesting) rate due to low market participation, continues to be a critical barrier to increasing beef meat production in Tanzania. Furthermore, econometric estimates showed that the key factors that positively influenced beef meat output at a 5% significance level (*P < 0*.*05*) included beef cattle population (inventory), beef cattle yield (carcass weight (kg) per head, and the number of beef cattle slaughtered, with elasticity coefficients of 0.146, 0.469, and 0.564, respectively). While the number of beef cattle exported positively influenced beef meat production at the 10% significance level (*P < 0*.*1*) with an elasticity coefficient of 0.028. Surprisingly, invested credit to agriculture (farm credits) and imported pure-bred beef cattle had a negative impact on beef meat output but were statistically insignificant at *P < 0*.*05*. The results of this study have implications as to what factors need to be addressed to further improve beef meat production, thereby reducing its reliance on imports. We suggest that the Tanzania government and policymakers need to establish balanced policies for beef farmers and appropriately manage them so that beef meat development can be induced, contributing to poverty reduction, food security, and economic development.

## 1. Introduction

The rapid food revolution around the world is evident in the increasing demand for livestock products, especially in developing countries, with livestock farming anchored amid the advancement of the rural economy and alleviation of poverty [1]. Livestock farming distributes almost half of the world’s agricultural products [2]. Despite advances in the development of alternative protein sources (plants, algae, etc.) the demand for high-quality meat proteins is increasing owing to the growth of the world’s population [3]. According to the Food and Agriculture Organization of the United Nations [4] meat (beef, mutton, pork, poultry, and goat meat) remains the core global protein source. Research conducted by Henchion *et al*. [5] indicates that in the coming years, animal protein demand is expected to increase. Meat and meat products are often over-consumed in the diets of both developing and developed countries [6]. Recently, consumers have shown great interest in foods formulated with ingredients that are considered to be beneficial to health or to reduce ingredients that are considered unsafe [7, 8]. The quantity and quality of beef meat and its nutritional value and health-promoting properties are becoming more and more important in conscious and reasonable human nutrition [9].

In Tanzania, 94% of total beef cattle herds are predominantly produced using traditional beef cattle farming (a free-range production system), while only 6% is produced using commercial (modern) beef cattle farming. The traditional beef cattle industry provides 95% of the country’s beef meat [10, 11]. The national beef cattle herd is dominated by indigenous beef cattle which are currently displaying low productivity, but they have much potential if feed, health, and breed improvements can be made [12]. The main breeds of beef cattle in the country include Tanzania Shorthorn Zebu (TSHZ) characterized by small size mature body weight (200–350 kg); Longhorn Cattle (LHC) such as the Ankole which is characterized by large matured body weight (500–730 kg); and the Boran which has a large bodyweight (500–800 kg) [12].

Moreover, in Tanzania, the beef meat industry is an extremely important component of the agri-food sector. It provides high-value protein in the nation’s diet; and contributes to food security [13]. Beef meat is the leading component of the red meat (beef, goat, and mutton meat) produced and consumed in Tanzania [12]. Industry analysis in the base year (2016–17), shows that beef meat accounted for almost 82% of total red meat while goats and mutton accounted for 14% and 4%, respectively [12]. Despite these facts, the beef meat industry faces a series of challenges that have harmed its production performance [12, 13]. Limited access to land for improving feed production, including grazing areas, high prevalence of animal diseases, poor access to farm credits, inadequate infrastructure, inadequate marketing system, weak beef cattle farmers’ organizations, inadequate technical support services, and the low genetic potential of local beef cattle breeds are the main barriers to increasing beef meat production and productivity [12–14].

Many years ago, the Tanzanian government recognized the importance and potential of the beef meat industry in alleviating poverty, enhancing food security, and creating employment, and made a clear commitment to improving it (overcoming the challenges) when it approved the National Livestock Policy (NLP) 2006 from the Agriculture and Livestock Policy of 1997 [12]. A key objective of the NLP (2006) is to “contribute to national food security through increased production, processing and marketing of livestock products (beef meat) to meet national nutritional requirements [15]. Priority technology interventions (strategies) for improving the beef meat industry included the following: (i) enhancing the quality and quantity of beef cattle feed resources through the introduction of improved forage crops and animal feed management practices, as well as increased access to existing grazing lands; (ii) increasing the productivity of indigenous beef cattle by changing the genetic composition through breed selection through crossbreeding and strengthening animal husbandry initiatives; and (iii) improving the quality and quantity of animal health services, as well as beef cattle farmers’ access to these services, through private and/or private-public partnerships to reduce young and adult stock mortality [12, 15]. These interventions (strategies) strive to achieve self-sufficiency in quality beef meat supply by increasing beef cattle production and productivity. The anticipated outcomes are consistent with the country’s development objectives, which include (i) reducing poverty; (ii) achieving food security and nutrition; (ii) promoting economic growth; (iv) promoting exports (beef meat); and (v) promoting industrialization [12, 15, 16]. Further to that, livestock sector analysis (LSA) [16] predicted that interventions for beef meat production on traditional farms and commercial ranches, as well as feedlot development, would result in a 52% increase in total red meat production (a growth of 742,524 tons) by 2022 to meet domestic and export demand.

However, despite the government’s efforts, these efforts have not been able to effectively meet the growing demand for beef meat. Looking ahead, due to Tanzania’s rapid population growth and increased income and demand for animal-derived foods, the projected increase in beef meat production (52%) will not be able to meet the 71% expected growth in consumption by 2022 (to 867,302 tons) [16]. This will result in a 17% deficit (124,778 tons) by 2022 beef meat production and consumption balance [16]. In addition, the Livestock Analysis (LSA) shows that under the business-as-usual (BAU) investment scenario, there will be large red meat (beef meat) supply gap in 15 years (by 2031–32), estimated at 1.7 million tons. This indicates that the projected domestic beef meat production in 2031 satisfies only 15% of the domestic consumption requirements [16]. This projected deficit will put upward pressure on beef meat prices, thereby affecting consumption in Tanzania. The estimated per capita meat consumption is 12 kg, which is still below the 50 kg recommended by the FAO [10, 12, 17]. The expected supply gap will still be driven by poorly existing animal genetics, health, and feed barriers. Furthermore, these barriers have led the beef cattle industry’s contribution to the national economy to be very low, accounting for only 5.9% of the national GDP. [10, 11, 16, 18]. Likewise, despite Tanzania having a large number of beef cattle, estimated to be 34.5 million, ranking third (3^rd^) in Africa and 11^th^ in the world, the country continues to import high-quality beef meat (over 700 metric tons per year) to meet the domestic demand for beef meat [12, 14, 17, 18]. This demonstrates that the relationship between the beef cattle population and beef meat production is weak, resulting in the limited availability of beef meat in the country. This reveals the potential growth of domestic and export demand for beef meat, which is an indication of an integrated enterprise (improvement)) in the beef meat industry. Thus, Tanzania needs to make more serious efforts (industry improvement) to narrow the supply-demand gap for local beef meat. According to Kibona and Yuejie [14], low market participation leads to low beef meat production. However, in addition to the number of beef cattle as a factor influencing beef meat production, other important factors such as (i) beef cattle yield (carcass weight per head); (ii) number of slaughtered beef cattle; (ii) invested credits to agriculture; (iii) imported pure and non-pure breeds of beef cattle; (iv) the total number of live beef cattle exported; and (iv) the policy implementation period, need to be investigated nationwide to improve beef meat production in Tanzania. Recently no research has explored the clear correlation between beef meat production and its influencing factors in Tanzania.

Developing countries with a high proportion of beef cattle production, such as Tanzania, should adopt innovative commercial strategies to utilize domestic and export business opportunities by increasing beef meat production [1]. Domestic and export shortages of beef meat supply indicate the potential of beef cattle agribusiness as a potential route for rural and national economic growth [1]. As a result, enhancing (improving) beef meat production is a potential strategy for improving rural livelihoods and the national economy. However, the implementation of the beef industry improvement strategy necessitates the government demonstrating a competitive level of knowledge on how beef meat production is affected by its determinants (influencing factors) [19].

Given the value of the beef meat industry in Tanzania, it is crucial to assess the fundamental reasons for and the direction of the relationship between beef meat output and its influencing factors such as beef cattle inventory (heads), the yield of beef cattle (carcass weight (hg) per head), the quantity of slaughtered beef cattle (heads), invested credits to agriculture (US$), the number of live beef cattle exported (heads), and quantity of pure-breed beef cattle imported (heads), as well as the implementation of national livestock policy of 2006. To the best of the authors’ knowledge, there is almost little or no assessment of the underlying reasons for and direction of the relationship between beef meat output and its influencing factors in the production processes during the period from 1990 to 2019, which this study aims to fill. Importantly, understanding such direction and relationship will aid in the development of effective action plans for enhancing beef meat production to achieve beef meat self-sufficiency, thereby reducing its reliance on imports. Similarly, increased beef meat production will significantly contribute to the country’s GDP and satisfy domestic and overseas market demand.

## 2. Materials and methods

### 2.1. The dataset and collection methods

The type of data obtained for the empirical analysis is secondary and publicly available time-series panel data for the period from 1990 to 2019. The data sources used in this study include the COMTRADE database, Food, and Agriculture Organization of the United Nations Statistical Database (FAOSTAT), and the National Bureau of Statistics (NBS). FAO (FAOSTAT), COMTRADE, and the National Bureau of Statistics (NBS) have committed to maintaining the best possible capacity to collect, process, validate, harmonize and analyze incoming data and generate and disseminate accurate and timely information on food and agriculture. Data are collected every year through questionnaires, submitted to countries by the teams published in FAOSTAT and COMTRADE databases regularly. Time series panel data (variables) collected from these sources include (i) beef cattle population (heads); (ii) beef meat production quantity (tons); (iii) yield of beef cattle (carcass weight (hg) per head); (iv) quantity of slaughtered beef cattle (heads); (v) invested credits to agriculture (US$); quantity of live beef cattle exported (heads); (vi) quantity of pure-breed beef cattle imported (heads); and (vii) new national livestock policy of 2006 (NLP 2006) implementation periods (dummy variables- 0 = years before national policy implementation, 1 = year with the policy implemented). The nominal values of credits invested in agriculture were adjusted to 2015 prices using the consumer price index (CPI) obtained from the NBS. In this study, beef meat production (tons) was the dependent variable, and the other variables were independent. The original time series dataset and its units are listed in S1 File.

### 2.2. Ethical considerations

This study was approved by the Jilin Agricultural University Graduate Research Ethics Committee in China. It was then submitted to and approved by the Ministry of Livestock and Fisheries (MLF) with reference number (AB.16/2020/01). The data collection process involved panel data gathering, therefore no human participants were involved; hence, consent was not obtained.

### 2.3. Data analysis

The study applied both descriptive statistics and econometric analytical models to analyze the time-series panel data. Descriptive statistical analysis methods such as line graphs, percentages, frequencies, mean values, and standard deviations are used to analyze the beef meat production quantity, beef cattle yield, beef cattle inventory, slaughtered heads, and extraction rate trends. An econometric analysis, a Cobb-Douglas production function model, using the Ordinary Least Squares (OLS) based estimator was used to analyze the factors that influenced beef meat production quantity trends in Tanzania from 1990 to 2019.

### 2.4. Econometric analytical model

This study was built on the Cobb-Douglas production function theory (model). The empirical analytical model was used to determine the fundamental factors of Tanzanian beef meat production for the period from 1990 to 2019. The theory (model) describes the relationship between production output (the amount of beef meat produced) and production inputs (influencing factors) [20]. The Cobb-Douglas Production function model mathematically estimates output (Q) as a function of aggregate input factors, Labour (L), and Capital (K) [20]. The equation for the production function can be simplified as:

$$Q=f\left(L^{\alpha }K^{\beta }\right)$$ (1)

Where Q stands for output, while *α* and *β* represent the elasticities of output for labor and capital [20]. The function is homogenous where *α* + *β* = 1 identifying constant returns to scale [21]. The parameters *α* and *β* can be estimated using Ordinary Least Squares (OLS) estimator.

For the case of this study, y (Q) stands for beef meat output (dependent variable), while independent variables (L and K) stand for factors influencing beef meat production such as beef cattle population; yield of beef cattle; quantity of slaughtered beef cattle; invested credits to agriculture (US$); quantity of live beef cattle exported; quantity of pure- breed of beef cattle imported; and new national livestock policy of 2006 (NLP 2006) implementation periods.

Applying the natural log on both sides of Eq 1 to ensure stationery, normality, and homoscedasticity, provides the specification of the regression model as follows:

$$In\left(Y\right)=\;\beta_{0}+\beta_{1}+In\left(x_{1}\right)+\beta_{2}In\left(x_{2}\right)+\beta_{3}In\left(x_{3}\right)+\beta_{4}In\left(x_{4}\right)+\beta_{5}In\left(x_{5}\right)+\beta_{6}In\left(x_{6}\right)+\beta_{7}x_{7})+\varepsilon$$ (2)

Here, “*In*” indicates that variables are in natural logarithmic form, *Y* denotes the output, β_0_ is an intercept (constant), β_1_,…, β_7_ are the coefficients to be estimated, and X_1_,…., and X_7_ represent the vectors of the explanatory variables, and ε is the error term.

The truncated Cobb-Douglas production function using the Ordinary Least Squares (OLS) estimator, for the influencing factors to determine whether there is an immediate or delayed effect on beef meat production quantity was specified as (see Eq 3);

$$\begin{array}{l} In\left(BeefMeatProduction\;\;Quantity\right)=\;\beta_{0}+\beta_{1}+In\left(Beef\;Cattle\;Population\right)+\beta_{2}In\left(Yield\;of\;Beef\;Cattle\right)\\ +\beta_{3}In\left(Quantity\;of\;Slaughtered\;Beef\;Cattle\right)+\beta_{4}In\left(Invested\;Credits\;to\;Agriculture\right)\\ +\beta_{5}In\left(Quantity\;of\;Beef\;Cattle\;Exported\right)+\beta_{6}In\left(Quantity\;of\;Pure-\;Breeds\;of\;Beef\;Cattle\;Imported\right)\\ +\beta_{7}(New\;National\;Livestock\;policy\;of\;2006\;\;Implementation\;Periods\\ +\varepsilon \; \end{array}$$ (3)

The descriptive statistics in the original form and the hypothesized sign effects of the independent variables (influencing factors) used in the regression model are shown in Table 1. The converted time-series dataset to the natural logarithm and its units are shown in S2 File.

**Table 1:** Descriptive statistics and expected sign effects of the independent variables on beef meat production quantity from 1990 to 2019.

Variables	Mean	Maximum	Minimum	Effects Sign
Beef Cattle Population (heads)	18,933,726.6 (4,930,577.06)*	27,821,063.0	13,046,835	+
Yield of Beef Cattle (carcass -hg /cattle)	1046.4 (135.65)*	1472.0	936	+
Slaughtered Beef Cattle (heads)	2,363,077.3 (520,569.8)*	3,260,987.0	1,800,000	+
Invested Credits to Agriculture (Mil.US$)	211.5 (251.16)*	639.3	0	+
Beef Cattle Exported (heads)	4308.3 (9,182.98)*	44768.0	0	-
Beef Cattle Imported (breeds)	489.0 (1907.8)*	10,386.0	0	+
National Livestock policy of 2006 Implementation Periods (dummy, 0, 1)	0.48 (0.5)*	1.0	0.0	±

*Numbers in parentheses the standard deviations.

## 3. Results and discussions

### 3.1. Beef cattle inventory, slaughtered beef cattle, and extraction rate

**Fig 1:**
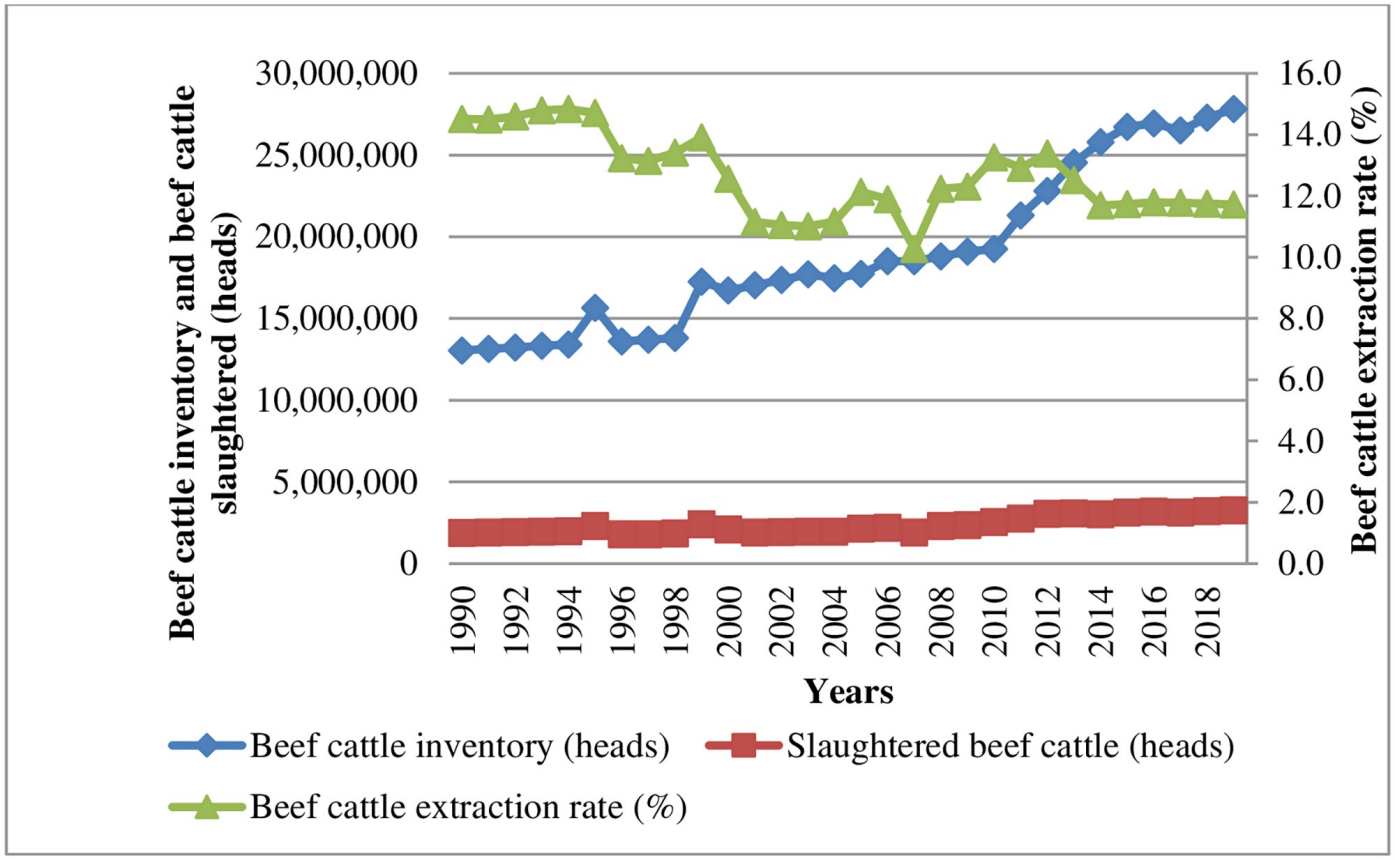
Beef cattle inventory, slaughtered beef cattle, and extraction rate.

Beef cattle inventory trend revealed changes in beef cattle stock (inventories) in Tanzania during a specific period, as shown in Fig 1 and Table 2. The analysis shows that beef cattle stocks (inventories) continued to increase from 1990 to 1995. It reached a historic peak of 15.7 million heads in 1995. Between 1995 and 1998, beef cattle inventories were clearly on the decline, with Tanzania’s beef cattle declining by 1.9 million. This is an important indicator of the failure of the Tanzanian beef cattle industry. This decline was affected by inadequate production techniques used by traditional beef cattle farmers because most of the total beef cattle herds are produced primarily by traditional beef cattle farming (a free-range production system) using poor production technologies [10, 12]. A sustained increase was observed again between 1999 and 2010, with beef cattle inventories increasing from 17.3 million to 19.3 million heads. A sharp increase in beef cattle inventories was observed from 2011 to 2019, reaching a peak of 27.9 million. This is an increase of 14.8 (53.05%) million between 1990 and 2019 (see Table 2). The sharp increase was due to the introduction of a new national livestock policy (NLP) in 2006 and the increase in land area allocated for grazing [15]. The national livestock policy (NLP) 2006 aimed to achieve meat self-sufficiency by increasing the production and productivity of beef cattle inventories, influenced by the increasing use of modern production techniques among beef farmers. The potential for growth of the beef cattle inventory in Tanzania is high because the country has favorable conditions and vast land, which can support the growth of the industry. There are approximately 60 million hectares of pasture suitable for grazing in Tanzania [11]. The future growth of the industry provides opportunities for both domestic and foreign investors to invest in the production and processing of beef meat for domestic and export markets.

**Table 2:** Changes in the overall structure of beef cattle inventory, slaughtered beef cattle, and extraction rate from 1990 to 2019.

years	Beef Cattle Inventory *(Million heads)*	Beef Cattle Slaughtered *(Million heads)*	Beef Cattle *Extraction Rate (%)*
1990	13.1	1.9	14.5
2019	27.9	3.3	11.7
**Total Changes**	14.8(53.05%)(+)	1.4(42.42%)(+)	2.8(-)

The number of beef cattle slaughtered in Tanzania from 1990 to 2019 is also shown in Fig 1 and Table 2. The analysis indicates that from 1990 to 2019, the annual quantity of slaughtered beef cattle reached the remarkable high of 3.3 million head from 1.9 million head, which is an increase of 1.4 (42.42%) million heads as indicated in Table 2. The rapid growth is mainly due to the continuous growth of beef meat production and domestic consumption, as well as the vigorous development of the slaughter and beef processing industry during the same period.

Further investigation revealed that the extraction rate of beef cattle inventory has been declining, as shown in Fig 1 and Table 2. In 1990, the extraction rate was 14.5%; by 2019, it had declined dramatically to 11.7% (see Table 2). This is a surprising finding, although the quantity of slaughtered beef cattle increased the average annual extraction rate decreased by 2.8% over this period. This indicates insufficient extraction of beef cattle inventory. The supply of beef cattle for meat production is low. According to Kibona and Yuejie [14], beef cattle farmers participate in the market by selling an average of only 5 beef cattle per year, which is very low for the maximum extraction of available beef cattle stocks. It is observed that the wealth protection and prestige felt by beef cattle farmers after accumulating a huge beef cattle herd exceeds market incentives, which jeopardizes the integration of beef cattle farmers into the organized market, resulting in a low extraction rate of beef cattle inventory [22]. Tanzania’s beef cattle industry still has great potential to promote beef meat production for domestic and international consumption: increasing farmers’ market participation to increase the extraction rate of beef cattle inventory is likely to be one of the best means to achieve this goal.

### 3.2. Beef meat production and carcass yield trends in Tanzania (1990–2019)

**Fig 2:**
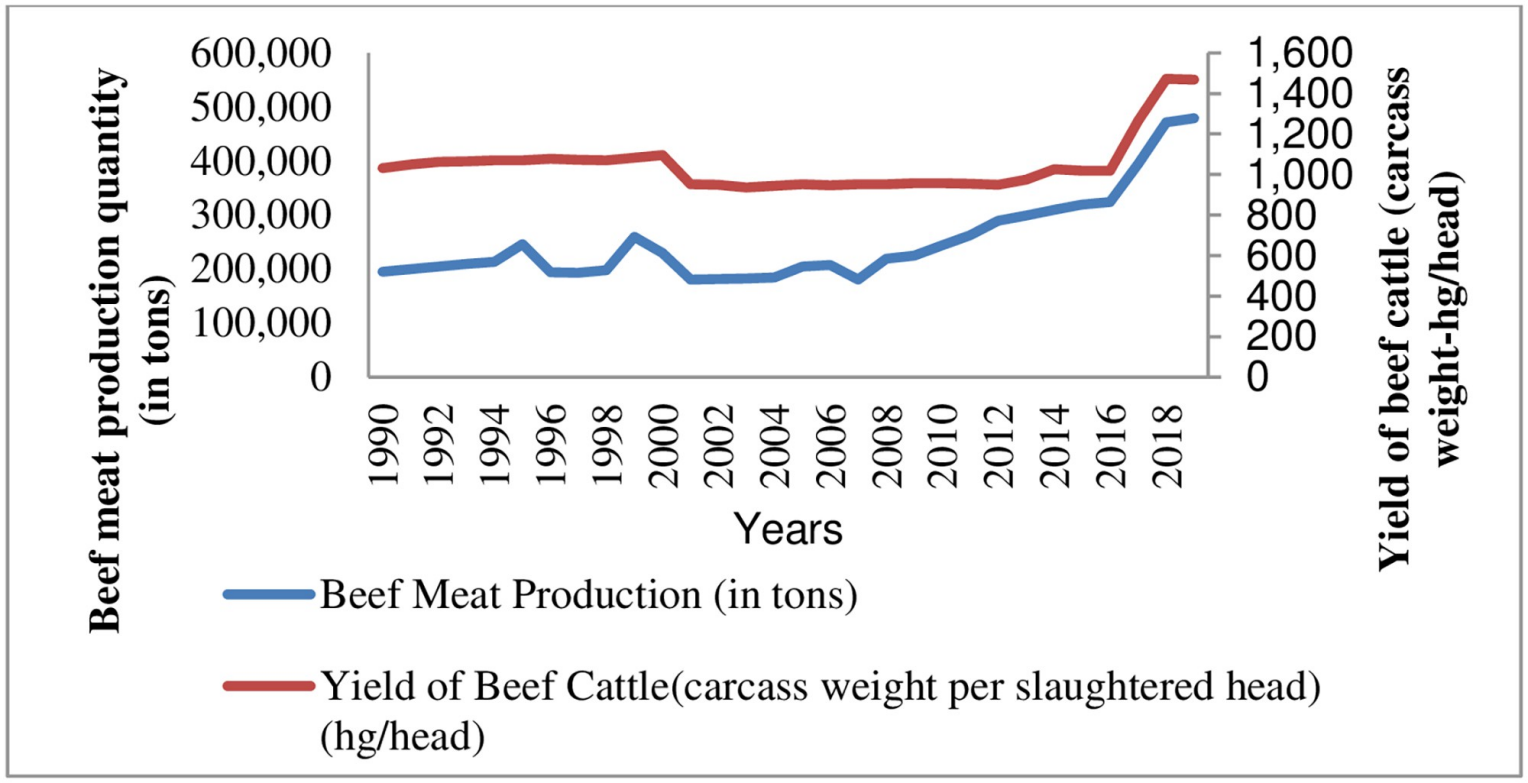
Beef meat production and carcass yield trends in Tanzania (1990–2019).

The analysis results show that Tanzania’s beef meat production increased by 283,871 tons (59.3%) between 1990 and 2019, as shown in Fig 2 and Table 3. Before the effective implementation of the new national livestock policy in 2006, beef meat production experienced a continuous decline and upward trend from 1990 to 2007. From 2008 to 2019, it further achieved an upward trend, reaching a significant peak of 479,071 tons. This increase was accompanied by a significant increase in beef cattle yield (carcass weight per slaughter head). The results further showed that beef cattle yield (carcass weight per head) increased from 1032 hectograms (hg) per head in 1990 to 1,469 hectograms (hg) per head in 2019, an increase of 437 (29.75%) hectograms (hg) per head, as shown in Table 3. As a result of the new national livestock policy of 2006, the yield of beef cattle has been increasing due to the adoption of beef cattle fattening technology recently introduced in the country [23, 24]. In addition, the findings also show that increased beef meat production was influenced by increasing trends in beef cattle inventories and slaughtered beef cattle during the stated period (Fig 1 and Table 2).

**Table 3:** Overall changes in beef production and carcass yield trends.

years	Beef Meat Production *(Tons)*	The yield of Beef Cattle (carcass weight/head) *(Hg/head)*
1990	195,200	1,032
2019	479,071	1,469
**Total Changes**	**283,871(59.3%) (+)**	**439(29.75%) (+)**

### 3.3. Econometric estimates of factors influencing beef meat production in Tanzania (1990–2019)

The parameter values estimated by the log-linear ordinary least squares (OLS) regression model are shown in Table 4. The model was estimated using SPSS V. 22 and goodness of fit statistics which shows that the model was significant at 5% level (*p < 0*.*05*) with R^2^ = 0.996 and Adjusted R^2^ = 0.995. The coefficient of determination denoted by R^2^ is a goodness-of-fit measure that indicates that independent variables account for about 99.6% of the rate of change in total beef meat production in Tanzania. The remaining 0.4% is the unexplained variation in beef meat production quantity which is captured by the error term (*ε*). This indicates that the independent variables in the model are explaining a large proportion of the variation in Tanzanian beef meat production. In general, the higher the R^2^, the better the model fits the data [25, 26].

**Table 4:** Estimates of log-log linear Ordinary Least Squares (OLS) parameters on the factors that influenced beef meat production in Tanzania from 1990 to 2019.

Key variables	Coefficients (β)	Std. Error
(*In)*Beef cattle population (heads)	0.146*	0.001
(*In*)Beef cattle yield (carcass weight/cattle (hg/head))	0.469*	0.001
(*In*)Slaughtered beef cattle (heads)	0.564*	0.010
(*In*)Invested credit to agriculture (million US$)	-0.065	0.000
(*In*)Beef cattle exported (heads)	0.028**	0.050
(*In*)Pure-breed beef cattle imported (heads)	-0.02	0.000
(*In*)Livestock policy of 2006 implementation periods (0, 1)	0.042	0.000
Constant	-9.21	0.006
R Squared (R^2^)	0.996(99.6%)	
Adjusted R squared (Adj. R^2^)	0.995(99.5%)	
F value	824.345*	

*Indicate significance level at 5% (P < 0.05) and

** at 10% (P < 0.10); (In)-indicates variables are in natural logarithm.

In economic verification, we checked whether the estimated value obtained meets the expected sign effect of the hypothesis (see Table 1). Therefore, using the results of the estimates indicated in Table 4, the equation representing Tanzania’s beef meat production (Iny_1_), depending on beef cattle population (Inx_1_); yield of beef cattle (carcass weight per head) (Inx_2_); quantity of slaughtered beef cattle (Inx_3_); Invested credits to agriculture (US$) (Inx_4_); quantity of live beef cattle exported (Inx_5_); quantity of pure- breeds of beef cattle imported (Inx_6_); and new national livestock policy of 2006 (NLP 2006) implementation periods (x_7_) is mathematically written as follows:

$$Iny_{1}=\;-0.921+0.146Inx_{1}+0.469Inx_{2}+0.564Inx_{3}-0.065Inx_{4}+0.028Inx_{5}-0.02Inx_{6}+0.042Inx_{7}$$ (4)

Hence; holding other factors unchanged (ceteris paribus) the coefficient estimates indicate that;

The beef cattle population (beef cattle inventory) had a positive effect on the variation of beef meat production quantity and was statistically significant at the 5% significance level. The positive effect indicates that if the beef cattle population increases by one percent (1%), beef meat production quantity increases on average by 0.146%. Similarly, [27, 28] denote that beef meat production is entirely dependent on the local beef cattle supply (beef cattle population). As a result, implementing strategies to increase the beef cattle population is critical if optimal beef meat production is to be achieved in Tanzania. Such strategies include, but are not limited to, disease control and feed resource availability. This is because the prevalence of animal diseases and feed availability are among the challenges confronting Tanzania’s beef cattle inventory growth [12].

As expected, the coefficient of the beef cattle yield (carcass weight per head) was positive and statistically significant at a 5% level. This shows that for every 1% increase in beef cattle yield, beef meat production quantity increases by 0.469%. The findings are similar to those of [29], who discovered that beef cattle yield (carcass weight per head) is positively and significantly related to an increase in beef meat output. As a result, beef cattle farmers in Tanzania should focus on strategies that increase beef cattle yield to increase beef meat production quantity. Bertelsen [30] identifies three factors associated with beef cattle yield: (i) beef cattle classes to be kept: heifers have a slightly higher yield than steers; (ii) feeding practices: the longer the beef cattle are fed, the better the results. Thus, farmers should consider feeding high-quality feed to beef cattle for a longer time to increase yield and quality, thereby promoting beef meat production in Tanzania [23]; and (iii) beef cattle breed type: beef meat cattle breeds typically have thinner hides and yield more than dairy cattle breeds. In general, beef cattle with more muscle tend to yield better because the muscle adds weight to the carcass.

The number of beef cattle slaughtered showed a positive impact on beef meat production quantity and it was significant at a 5% significance level. This implies that beef meat production quantity increased by 0.564% for every one percent increase in the number of beef cattle slaughtered. This result was similar to the findings of [28], which noted that a rise in the number of beef cattle slaughtered fueled positive change in the Chinese beef meat industry. However, there has been a response from the feedlot sector, as well as no shortage of funding and governmental policies in the slaughter and allied beef cattle supply industries [28]. Thus, beef cattle supply for meat production should be promoted in Tanzania by creating incentives that encourage market participation among traditional beef cattle farmers. The higher the market participation the higher the supply of beef cattle for slaughter, hence higher beef meat production. Nevertheless, farmers in the traditional beef cattle sector, which occupy 94% of beef cattle herds stock in Tanzania [10, 12], are well known for their low market participation [14]. Therefore, to overcome the situation, a study by Kibona and Yuejie [14] suggested that the Tanzania government should establish a strategic cooperative to function not only as a communication channel for farm credits, price, market information, and training on commercial farming but also to assist farmers in selection of profitable markets.

Farm credit is important for investing in beef cattle production and marketing activities, thus boosting beef cattle productivity, which increases the tendency to supply more beef cattle to the market for beef meat production [22]. This study, however, discovered that invested credit to agriculture negatively influenced the quantity of beef meat produced in Tanzania over the stated period, but was statistically insignificant at the 5% significance level. In this regard, if the amount of credit invested in agriculture increases by 1%, the beef meat output decreases by 0.065%. This finding contradicts [22], but is similar to Musah et al. [31], who discovered a negative impact of credit on beef meat output. The negative findings of this study imply that beef cattle farmers do not use farm credit as capital in production, thereby decreasing the level of beef meat production. Additionally, farmers usually reallocate credit to non-beef cattle business activities and misuse it, impeding beef cattle business development and limiting beef meat production quantity. As a result, farmers require appropriate and convenient credit-securing arrangements as well as assistance in the establishment of a marketing system that will add value to the beef cattle business to boost beef meat production in Tanzania [22].

Furthermore, even though exporting live beef cattle reduces beef cattle supply to the market for beef meat production, the finding of this study revealed a positive influence on beef meat production quantity in Tanzania, which was statistically significant at a 10% significance level. This means that if the volume of beef cattle exported increases by 1%, beef meat production increases by 0.028%. In addition, this means that reinvesting the value of beef cattle exports (income) in beef cattle production and marketing activities increases beef cattle productivity and surplus beef cattle for slaughter, thereby increasing beef meat production quantity. As a result, a favorable business environment for the export of live beef cattle should be created to generate income (foreign exchange income-earning) as the capital for the beef cattle business.

Likewise, the import of purebred beef cattle and the implementation of livestock policies had a negative and positive effect, respectively, but the correlation with beef meat production quantity is not significant at the 5% and 10% significance levels, respectively. This result appears to contradict [29] who demonstrated the beneficial impact of genetic manipulation (crossbreeding purebred) on increasing meat production output on the farm. As a result, increased efforts to implement livestock policy are recommended. Besides that, beef cattle breeds that are imported into Tanzania for crossbreeding must be compatible and adaptable to the Tanzanian environment, thereby improving hybrid vigor and promoting beef meat production.

## 4. Conclusion

The value of beef meat production in Tanzania was positively influenced by beef cattle population, beef cattle yield (carcass weight/cattle (hg/head), slaughtered beef cattle (heads), beef cattle exported (heads), and implementation of livestock policy of 2006 periods, while factors which were negatively influencing beef meat production was invested credit in agriculture and pure-breed beef cattle imported. Beef meat is important for global food security because it accounts for 17% of global calorie consumption and 33% of global protein consumption [32–34]. As a result, to increase the value of beef meat production in Tanzania, the government should develop effective action plans and industry support policies to promote beef cattle quality, yield, and productivity. Moreover, such policies should focus on creating incentives for farmers to participate in the market, which promotes beef cattle supply in the market (increased beef cattle extraction rate), resulting in increased beef meat production. Furthermore, emphasizing proper utilization of farm credit (invested credit in agriculture) through providing farmers with suitable and convenient credit-securing arrangements will add value to the beef cattle business, thereby boosting beef meat production.

## Supporting information

S1 File (DOCX)

S2 File (DOCX)

S1 Dataset (SAV)
