# Supplementary material for: Factors that influence beef meat production in Tanzania. A Cobb-Douglas production function estimation approach
Source: PLoS One. 2022 Aug 12;17(8):e0272812. doi: 10.1371/journal.pone.0272812 (PMC9374255; doi:10.1371/journal.pone.0272812)
Supplement: S1 File — (DOCX) [file pone.0272812.s001.docx]

**The original time series dataset and its units are listed in supporting information Table 1.**

**Table 1. Original dataset**

| Year | Beef Prod. | Cattle Population. | Cattle yield | Slaughtered Cattle | Credits Invested | Cattle Export | Breeds import | Policy (2006) |
| --- | --- | --- | --- | --- | --- | --- | --- | --- |
|  | **Tons** | **Heads** | **Hg/head** | **Heads** | **Mil US$** | **Heads** | **Heads** | **(0,1)** |
|  | ***y_1_*** | ***x_1_*** | ***x_2_*** | ***x_3_*** | ***x_4_*** | ***x_5_*** | ***x_6_*** | ***x_7_*** |
| 1990 | 195,200 | 13,046,835 | 1,032 | 1,892,000 | 0 | 0 | 20 | 0 |
| 1991 | 200,000 | 13,138,162 | 1,050 | 1,905,000 | 0 | 0 | 0 | 0 |
| 1992 | 205,000 | 13,230,100 | 1,062 | 1,930,000 | 0 | 0 | 0 | 0 |
| 1993 | 210,000 | 13,322,700 | 1,066 | 1,970,000 | 0 | 0 | 0 | 0 |
| 1994 | 213,000 | 13,416,000 | 1,070 | 1,990,000 | 0 | 0 | 0 | 0 |
| 1995 | 246,000 | 15,644,800 | 1,070 | 2,300,000 | 0 | 0 | 4 | 0 |
| 1996 | 194,000 | 13,604,500 | 1,078 | 1,800,000 | 0 | 0 | 4 | 0 |
| 1997 | 193,000 | 13,699,700 | 1,072 | 1,800,000 | 0 | 1,375 | 42 | 0 |
| 1998 | 198,000 | 13,795,600 | 1,070 | 1,850,000 | 0 | 480 | 57 | 0 |
| 1999 | 260,000 | 17,250,762 | 1,083 | 2,400,000 | 0 | 89 | 56 | 0 |
| 2000 | 230,000 | 16,713,000 | 1,095 | 2,100,000 | 26.8 | 372 | 156 | 0 |
| 2001 | 181,000 | 17,037,000 | 953 | 1,900,000 | 44.4 | 77 | 0 | 0 |
| 2002 | 182,000 | 17,367,000 | 948 | 1,920,000 | 101.1 | 580 | 106 | 0 |
| 2003 | 182,500 | 17,704,000 | 936 | 1,950,000 | 0 | 1,997 | 69 | 0 |
| 2004 | 184,000 | 17,472,118 | 944 | 1,950,000 | 0 | 3,610 | 30 | 0 |
| 2005 | 204,520 | 17,719,092 | 951 | 2,150,000 | 0 | 2,103 | 156 | 0 |
| 2006 | 208,046 | 18,500,000 | 946 | 2,200,000 | 213.2 | 4,383 | 35 | 1 |
| 2007 | 180,629 | 18,500,000 | 951 | 1,900,000 | 239.7 | 2,879 | 116 | 1 |
| 2008 | 218,976 | 18,800,000 | 952 | 2,300,000 | 431.3 | 2,484 | 86 | 1 |
| 2009 | 225,178 | 19,100,000 | 958 | 2,350,000 | 353.8 | 2,400 | 27 | 1 |
| 2010 | 243,943 | 19,245,648 | 957 | 2,550,000 | 490.5 | 1,091 | 74 | 1 |
| 2011 | 262,606 | 21,300,000 | 955 | 2,750,000 | 580.3 | 2,823 | 671 | 1 |
| 2012 | 289,835 | 22,800,000 | 950 | 3,050,000 | 593.1 | 899 | 161 | 1 |
| 2013 | 299,581 | 24,531,672 | 976 | 3,070,000 | 603.0 | 2,502 | 150 | 1 |
| 2014 | 309,353 | 25,800,000 | 1,026 | 3,014,575 | 639.3 | 1,311 | 76 | 1 |
| 2015 | 319,112 | 26,713,644 | 1,018 | 3,135,458 | 589.7 | 12,990 | 2,060 | 1 |
| 2016 | 323,775 | 26,935,923 | 1,019 | 3,177,658 | 508.8 | 1,803 | 69 | 1 |
| 2017 | 394,604 | 26,519,776 | 1,264 | 3,121,863 | 509. | 21,751 | 23 | 1 |
| 2018 | 471,692 | 27,282,702 | 1,472 | 3,204,778 | 1420.9 | 16,481 | 37 | 1 |
| 2019 | 479,071 | 27,821,063 | 1,469 | 3,260,987 | 0.0 | 44,768 | 10,386 | 1 |

**The converted time-series dataset to the natural logarithm and its units are shown in the supporting information Table 2.**

**Table 3. Dataset converted into natural logarithm**

| Year | Beef Prod. | Cattle Population. | Cattle yield | Slaughtered Cattle | Credits Invested | Cattle Export | Breeds import | Policy (2006) |
| --- | --- | --- | --- | --- | --- | --- | --- | --- |
|  | **Tons** | **Heads** | **Hg/head** | **Heads** | **Mil US$** | **Heads** | **Heads** | **(0,1)** |
|  | ***Lny_1_*** | ***Lnx_1_*** | ***Lnx_2_*** | ***Lnx_3_*** | ***Lnx_4_*** | ***Lnx_5_*** | ***Lnx_6_*** | ***x_7_*** |
| 1990 | 12.18 | 16.38 | 6.94 | 14.45 | 0.00 | 0.00 | 3.00 | 0 |
| 1991 | 12.21 | 16.39 | 6.96 | 14.46 | 0.00 | 0.00 | 0.00 | 0 |
| 1992 | 12.23 | 16.40 | 6.97 | 14.47 | 0.00 | 0.00 | 0.00 | 0 |
| 1993 | 12.25 | 16.40 | 6.97 | 14.49 | 0.00 | 0.00 | 0.00 | 0 |
| 1994 | 12.27 | 16.41 | 6.98 | 14.50 | 0.00 | 0.00 | 0.00 | 0 |
| 1995 | 12.41 | 16.57 | 6.98 | 14.65 | 0.00 | 0.00 | 1.39 | 0 |
| 1996 | 12.18 | 16.43 | 6.98 | 14.40 | 0.00 | 0.00 | 1.39 | 0 |
| 1997 | 12.17 | 16.43 | 6.98 | 14.40 | 0.00 | 7.23 | 3.74 | 0 |
| 1998 | 12.20 | 16.44 | 6.98 | 14.43 | 0.00 | 6.17 | 4.04 | 0 |
| 1999 | 12.47 | 16.66 | 6.99 | 14.69 | 0.00 | 4.49 | 4.03 | 0 |
| 2000 | 12.35 | 16.63 | 7.00 | 14.56 | 3.29 | 5.92 | 5.05 | 0 |
| 2001 | 12.11 | 16.65 | 6.86 | 14.46 | 3.79 | 4.34 | 0.00 | 0 |
| 2002 | 12.11 | 16.67 | 6.85 | 14.47 | 4.62 | 6.36 | 4.66 | 0 |
| 2003 | 12.11 | 16.69 | 6.84 | 14.48 | 0.00 | 7.60 | 4.23 | 0 |
| 2004 | 12.12 | 16.68 | 6.85 | 14.48 | 0.00 | 8.19 | 3.40 | 0 |
| 2005 | 12.23 | 16.69 | 6.86 | 14.58 | 0.00 | 7.65 | 5.05 | 0 |
| 2006 | 12.25 | 16.73 | 6.85 | 14.60 | 5.36 | 8.39 | 3.56 | 1 |
| 2007 | 12.10 | 16.73 | 6.86 | 14.46 | 5.48 | 7.97 | 4.75 | 1 |
| 2008 | 12.30 | 16.75 | 6.86 | 14.65 | 6.07 | 7.82 | 4.45 | 1 |
| 2009 | 12.32 | 16.77 | 6.86 | 14.67 | 5.87 | 7.78 | 3.30 | 1 |
| 2010 | 12.40 | 16.77 | 6.86 | 14.75 | 6.20 | 6.99 | 4.30 | 1 |
| 2011 | 12.48 | 16.87 | 6.86 | 14.83 | 6.36 | 7.95 | 6.51 | 1 |
| 2012 | 12.58 | 16.94 | 6.86 | 14.93 | 6.39 | 6.80 | 5.08 | 1 |
| 2013 | 12.61 | 17.02 | 6.88 | 14.94 | 6.40 | 7.82 | 5.01 | 1 |
| 2014 | 12.64 | 17.07 | 6.93 | 14.92 | 6.46 | 7.18 | 4.33 | 1 |
| 2015 | 12.67 | 17.10 | 6.93 | 14.96 | 6.38 | 9.47 | 7.63 | 1 |
| 2016 | 12.69 | 17.11 | 6.93 | 14.97 | 6.23 | 7.50 | 4.23 | 1 |
| 2017 | 12.89 | 17.09 | 7.14 | 14.95 | 6.23 | 9.99 | 3.14 | 1 |
| 2018 | 13.06 | 17.12 | 7.29 | 14.98 | 7.26 | 9.71 | 3.61 | 1 |
| 2019 | 13.08 | 17.14 | 7.29 | 15.00 | 0.00 | 10.71 | 9.25 | 1 |
