# Supplementary material for: Factors that influence beef meat production in Tanzania. A Cobb-Douglas production function estimation approach
Source: PLoS One. 2022 Aug 12;17(8):e0272812. doi: 10.1371/journal.pone.0272812 (PMC9374255; doi:10.1371/journal.pone.0272812)
Supplement: S2 File — (DOCX) [file pone.0272812.s002.docx]

**The converted time-series dataset to the natural logarithm and its units are shown in the supporting information Table 2.**

**Table 3. Dataset converted into natural logarithm**

| Year | Beef Prod. | Cattle Population. | Cattle yield | Slaughtered Cattle | Credits Invested | Cattle Export | Breeds import | Policy (2006) |
| --- | --- | --- | --- | --- | --- | --- | --- | --- |
|  | **Tons** | **Heads** | **Hg/head** | **Heads** | **Mil US$** | **Heads** | **Heads** | **(0,1)** |
|  | ***Lny_1_*** | ***Lnx_1_*** | ***Lnx_2_*** | ***Lnx_3_*** | ***Lnx_4_*** | ***Lnx_5_*** | ***Lnx_6_*** | ***x_7_*** |
| 1990 | 12.18 | 16.38 | 6.94 | 14.45 | 0.00 | 0.00 | 3.00 | 0 |
| 1991 | 12.21 | 16.39 | 6.96 | 14.46 | 0.00 | 0.00 | 0.00 | 0 |
| 1992 | 12.23 | 16.40 | 6.97 | 14.47 | 0.00 | 0.00 | 0.00 | 0 |
| 1993 | 12.25 | 16.40 | 6.97 | 14.49 | 0.00 | 0.00 | 0.00 | 0 |
| 1994 | 12.27 | 16.41 | 6.98 | 14.50 | 0.00 | 0.00 | 0.00 | 0 |
| 1995 | 12.41 | 16.57 | 6.98 | 14.65 | 0.00 | 0.00 | 1.39 | 0 |
| 1996 | 12.18 | 16.43 | 6.98 | 14.40 | 0.00 | 0.00 | 1.39 | 0 |
| 1997 | 12.17 | 16.43 | 6.98 | 14.40 | 0.00 | 7.23 | 3.74 | 0 |
| 1998 | 12.20 | 16.44 | 6.98 | 14.43 | 0.00 | 6.17 | 4.04 | 0 |
| 1999 | 12.47 | 16.66 | 6.99 | 14.69 | 0.00 | 4.49 | 4.03 | 0 |
| 2000 | 12.35 | 16.63 | 7.00 | 14.56 | 3.29 | 5.92 | 5.05 | 0 |
| 2001 | 12.11 | 16.65 | 6.86 | 14.46 | 3.79 | 4.34 | 0.00 | 0 |
| 2002 | 12.11 | 16.67 | 6.85 | 14.47 | 4.62 | 6.36 | 4.66 | 0 |
| 2003 | 12.11 | 16.69 | 6.84 | 14.48 | 0.00 | 7.60 | 4.23 | 0 |
| 2004 | 12.12 | 16.68 | 6.85 | 14.48 | 0.00 | 8.19 | 3.40 | 0 |
| 2005 | 12.23 | 16.69 | 6.86 | 14.58 | 0.00 | 7.65 | 5.05 | 0 |
| 2006 | 12.25 | 16.73 | 6.85 | 14.60 | 5.36 | 8.39 | 3.56 | 1 |
| 2007 | 12.10 | 16.73 | 6.86 | 14.46 | 5.48 | 7.97 | 4.75 | 1 |
| 2008 | 12.30 | 16.75 | 6.86 | 14.65 | 6.07 | 7.82 | 4.45 | 1 |
| 2009 | 12.32 | 16.77 | 6.86 | 14.67 | 5.87 | 7.78 | 3.30 | 1 |
| 2010 | 12.40 | 16.77 | 6.86 | 14.75 | 6.20 | 6.99 | 4.30 | 1 |
| 2011 | 12.48 | 16.87 | 6.86 | 14.83 | 6.36 | 7.95 | 6.51 | 1 |
| 2012 | 12.58 | 16.94 | 6.86 | 14.93 | 6.39 | 6.80 | 5.08 | 1 |
| 2013 | 12.61 | 17.02 | 6.88 | 14.94 | 6.40 | 7.82 | 5.01 | 1 |
| 2014 | 12.64 | 17.07 | 6.93 | 14.92 | 6.46 | 7.18 | 4.33 | 1 |
| 2015 | 12.67 | 17.10 | 6.93 | 14.96 | 6.38 | 9.47 | 7.63 | 1 |
| 2016 | 12.69 | 17.11 | 6.93 | 14.97 | 6.23 | 7.50 | 4.23 | 1 |
| 2017 | 12.89 | 17.09 | 7.14 | 14.95 | 6.23 | 9.99 | 3.14 | 1 |
| 2018 | 13.06 | 17.12 | 7.29 | 14.98 | 7.26 | 9.71 | 3.61 | 1 |
| 2019 | 13.08 | 17.14 | 7.29 | 15.00 | 0.00 | 10.71 | 9.25 | 1 |
